# Modeling Pressure‐Dependent Wave and Vessel Compliance in the Brain Following Middle Cerebral Artery Occlusion

**DOI:** 10.1111/micc.70049

**Published:** 2026-01-26

**Authors:** Erin Zhao, Jared Barber, Shomita S. Mathew‐Steiner, Savita Khanna, Chandan K. Sen, Julia Arciero

**Affiliations:** ^1^ Department of Mathematical Sciences Indiana University Indianapolis Indianapolis Indiana USA; ^2^ McGowan Institute for Regenerative Medicine University of Pittsburgh School of Medicine Pittsburgh Pennsylvania USA

**Keywords:** compliance, leptomeningeal collateral arteries, mathematical model, middle cerebral artery occlusion, pressure‐dependent wave, stroke

## Abstract

**Objective:**

This study demonstrates the impact of alterations in pressure, vascular compliance, arterial pulsatility, and autoregulation on tissue perfusion following middle cerebral artery (MCA) occlusion using mathematical modeling.

**Methods:**

Our previous mathematical model of the cerebral circulation is expanded to include vessel compliance and pulsatility of blood flow. An experimentally‐obtained pressure waveform is used as an incoming boundary condition to simulate the effects of vascular compliance and pulsatility of flow on perfusion following MCA occlusion. The waveform is adjusted to model the effects of elevated mean arterial pressure.

**Results:**

Increased distensibility reduces the amplitude of oscillations in the time‐dependent pressure solutions, whereas decreased distensibility produces more variation in these pressures. Occlusion significantly alters the magnitude of flow changes when incoming pressure is varied. The addition of the pulsatile pressure boundary condition and capacitances in the arteries and veins shifts the autoregulation plateau to higher pressures.

**Conclusions:**

This study reveals how changes in incoming pressure affect compensatory responses to ischemic stroke caused by MCA occlusion. Boundary conditions corresponding to elevated mean arterial pressures are associated with lower degrees of ischemia, an improvement that is supported by changes in autoregulation patterns following occlusion. The results also demonstrate how increases in arterial stiffness associated with aging can inhibit the ability of the vasculature to accommodate pulsatile flow by analyzing resulting patterns such as larger amplitudes of pressure and flow oscillations in the microcirculation. The study provides a foundation for modeling the relationships among vessel compliance, arterial blood pressure, and cerebrovascular conditions.

**Abbreviations**
A: activation
a: anterior branch
ACA: anterior cerebral artery
ACA1: pre‐communicating ACA
ACA2: post‐communicating ACA
ACoA: anterior communicating artery
BA: basilar artery
CAP: capillaries
COL: collateral arteries
D: diameter
Dy: diastolic pressure
ICA: internal carotid artery
LA: large arterioles
LV: large venules
m: middle branch
MAP: mean arterial pressure
MCA: middle cerebral artery
p: posterior branch
*P*_a_
: incoming arterial pressure
PCA: posterior cerebral artery
PCA1: pre‐communicating PCA
PCA2: post‐communicating PCA
PCoA: posterior communicating artery
PO_2_
: partial pressure of oxygen
Q: flow
S: systolic pressure
SA: small arterioles
SV: small venules
V: veins
VS: venous sinuses

## 1 Introduction

Blood flow and pressure in large arteries follow time‐dependent pulsatile patterns due to the effects of the cardiac cycle [1]. However, blood flow in the microcirculation must be maintained at a relatively steady rate in order to adequately meet tissue metabolic demand and limit pressure exposure in more fragile vessels such as capillaries [2]. In these smaller vessel regions, more continuous flow is attained by the mechanical properties of the cardiovascular system that dampen flow variability [3]. The volume elasticity of large arteries [1] converts changes in flow and pressure during the systolic phase of the heartbeat into volume discharge during diastole to maintain relatively constant perfusion to the microcirculation [4]. Pulse pressure, which is the difference between the systolic and diastolic pressures, quantifies the pulsatile component of blood pressure by assigning a measure to the magnitude of variations [5]. Hypertension and aging are associated with decreased arterial elasticity that reduces the ability of the circulatory system to accommodate pulsatile flow [6, 7, 8, 9, 10, 11]. This increased arterial stiffness and the resulting changes in pressure pulsatility are associated with pathological conditions common in advanced age, such as kidney disease and cognitive impairment, and are also considered predictive risk factors for cardiovascular morbidity and mortality [2, 5, 10, 12, 13].

Since the brain is a high‐flow, low‐impedance organ, pulsatile flow throughout the arterial tree is more pronounced in the brain than in the periphery [2]. Arterial stiffening further increases the pulsatile load of the cerebral circulation, which can result in the transmission of pulsations to the distal microcirculation and potentially expose capillaries to damaging levels of pressure pulsatility [2, 11, 14, 15]. This phenomenon is thought to hasten the progression of cerebral vascular diseases such as stroke [15], which is the second most prevalent cause of mortality and the third most common cause of disability worldwide [16]. In general, a stroke may occur due to stenosis or occlusion of large cerebral arteries [17]. A study of uncontrolled hypertensive subjects found that both elevated pulse pressure and mean arterial pressure were associated with a higher incidence of ischemic stroke [5]. Therefore, a study examining the effects of incoming perfusion pressure on cerebrovascular responses following vessel occlusion is useful and applicable to understanding the pathophysiology of stroke. Identifying how blood flow compensation, oxygen levels, and flow distribution are impacted by variations in the arterial pressure waveform could also lead to improved targeted treatments for populations susceptible to stroke such as hypertensive and/or elderly individuals.

The ability of arteries to accommodate pulsatile flow is frequently represented by Windkessel models, which account for the volume elasticity of vessels using compliance [15, 18, 19, 20, 21, 22]. These models consist of a combination of both resistance and compliance elements, where resistance is typically determined by Poiseuille's Law and arterial compliance is given by the ratio of the change in vessel volume to change in pressure [1]. Since the effects of pulsatile flow are significant in the brain, arterial compliance is thought to be an essential element of cerebral blood flow regulation; hemodynamic responses to changes in pressure amount to more than just changes in vascular tone by autoregulation [4].

Previous mathematical models have used this approach to gain an improved understanding of cerebral blood flow and have demonstrated the importance of compliance in pressure and flow regulation. Chan et al. [4] used a two‐element Windkessel model to analyze the contributions of cerebral resistance and compliance to blood flow velocity responses following drug‐induced (acute) changes in blood pressure and compared the Windkessel model to a model containing only resistances. They showed that integrating both resistance and compliance in the Windkessel model provided a better fit to flow velocity data. They concluded that the flow‐buffering effects of volume loading in capacitive arteries may play a major role in maintaining steady microvascular flow. Tzeng et al. [23] also highlighted the importance of compliance in cerebral blood flow by showing that changes in flow following a perturbation in blood pressure were primarily due to Windkessel properties. Both models focused on a signal processing approach, and thus time‐varying components such as active autoregulation were not assessed.

The regulation of cerebral pressure and blood flow depends on the interactions between mechanical properties of arteries and the modification of vascular tone. Unfortunately, there are no definitive methods for measuring cerebral autoregulation, making it difficult to infer its role in cerebrovascular dynamics [23]. However, several mathematical models have attempted to uncover this role. For example, Lakin et al. [24] included autoregulation in a lumped parameter model of whole‐body physiology to study changes in intracranial pressure dynamics with a pulsatile cardiac forcing function. Responses to orthostatic forces, cardiac arrest, and hemorrhagic shock were assessed. Resistance related flow to pressure drops along vessel compartments while compliance related differences between intraluminal pressure and the surrounding intracranial pressure to volume adjustments. Although autoregulation was used to simulate vessel constriction and dilation by adjusting the fluidities, it depended only on pressure values. The study in Ursino and Lodi [25] also combined principles of cerebral autoregulation and Windkessel effects to analyze their relationships to blood volume and intracranial pressure, where autoregulation was based on changes in cerebral blood flow relative to a basal level.

Mathematical modeling of the factors influencing cerebral autoregulation, in combination with the Windkessel properties of vessels, can potentially clarify how both autoregulation and compliance regulate responses to events such as vessel occlusion. To investigate the effects of internal carotid artery stenosis and middle cerebral artery occlusion, Ursino and Giannessi [26] developed a model of the Circle of Willis and the regions supplied by the main cerebral arteries. The arterial–arteriolar circulation in each region consisted of resistance and compliance elements which were modified by mechanisms including CO_2_ reactivity and autoregulation based on changes in the local blood flow level. Although regulatory responses to occlusion were studied, a constant arterial pressure was assumed, so the role of blood flow pulsatility was not considered.

These models that combined Windkessel effects with autoregulation mechanisms each focused on only one aspect influencing autoregulation—either deviations in blood pressure or flow. However, there are several factors that simultaneously contribute to autoregulation, which should be studied in combination to obtain a more complete picture of cerebrovascular responses to occlusion. The study by Zhao et al. [27] considered multiple components that influence autoregulation in arterioles and collaterals of the primary regions of the brain to investigate the effects of middle cerebral artery (MCA) occlusion but did not include the effects of pulsatility or vessel compliance. Large artery stiffness and pressure pulsatility are both associated with disturbances in microvascular function and impairments in the local regulation of blood flow [2], which necessitates a model that can effectively combine influential components of blood flow regulation and the effects of arterial compliance on pulsatility.

The present study incorporates several factors that influence autoregulation, including pressure, blood flow levels, and metabolic (oxygen) demand, into a model of the cerebral circulation consisting of both resistance and compliance components. Interactions between compliance and pulsatility are examined by implementing a pressure waveform representing blood flow entering from the carotid artery as the incoming boundary condition. This boundary condition is adjusted to assess the effects of an elevated mean arterial pressure (MAP), a condition that frequently occurs with stroke. The study aims to provide a more thorough understanding of the impact of alterations in pressure, vascular compliance, arterial pulsatility, and autoregulation on perfusion following cerebrovascular occlusion. Using mathematical models to reveal the most crucial components needed to maintain sufficient perfusion can ultimately provide insight into the management and treatment of ischemic stroke.

## 2 Materials and Methods

### 2.1 Vascular Network

**FIGURE 1:**
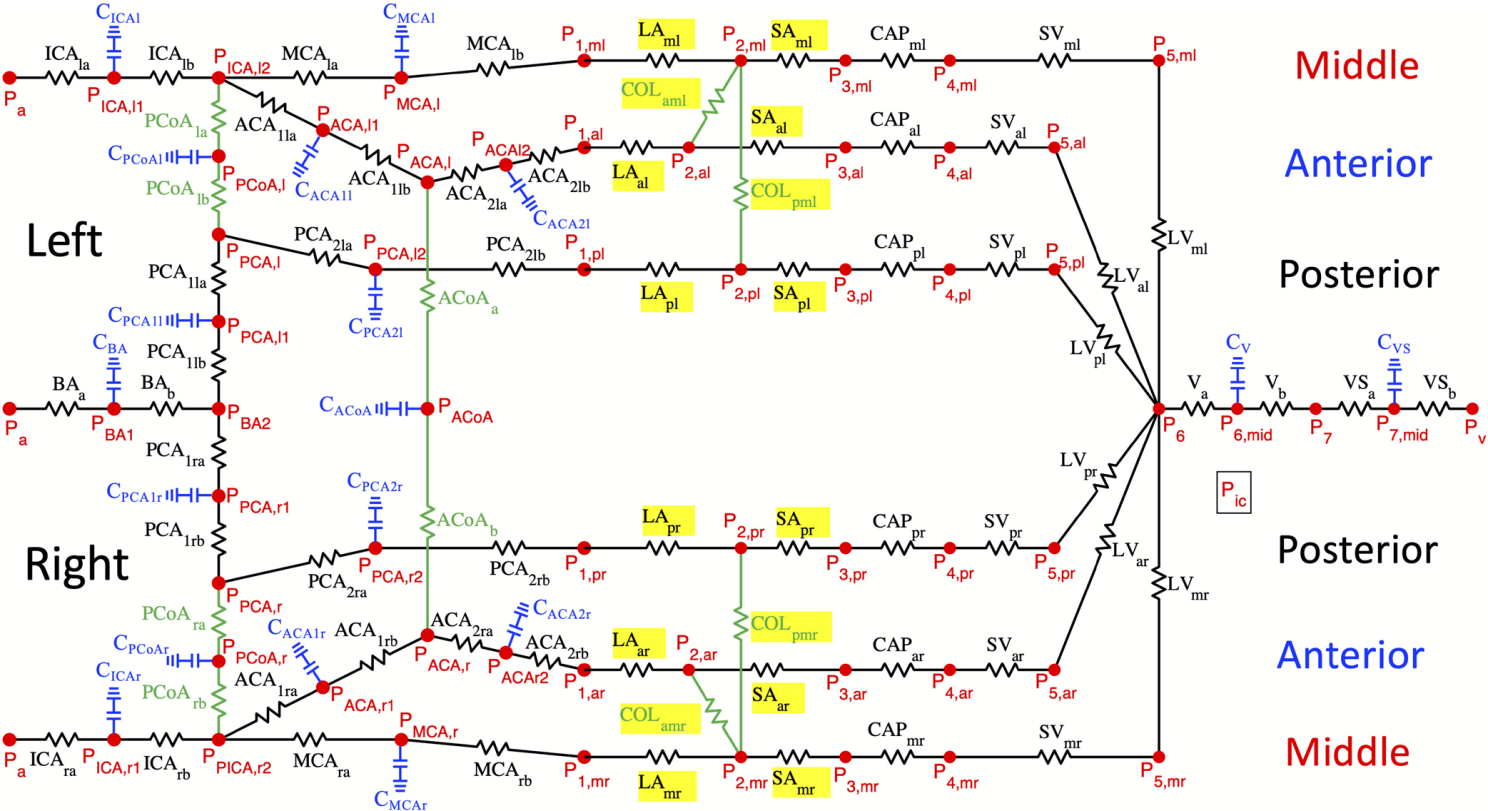
Diagram of the cerebral vasculature indicating the arteries and veins with associated capacitances. The arrangement of the vascular network is based on the structure in human brain with a complete Circle of Willis. Compartments of vessels are represented by resistors. The arteries, veins, and venous sinuses have associated capacitances (shown in blue) and are therefore divided into resistances *a* and *b*. Blood flow enters the brain through the internal carotid arteries (ICA) and basilar artery (BA) and is then supplied to different regions by the middle cerebral artery (MCA), anterior cerebral artery (ACA), and posterior cerebral artery (PCA). In the ACA and PCA, subscript 1 denotes the pre‐communicating portion of the artery and subscript 2 denotes the post‐communicating portions. Vessels that serve as collaterals are shown in green. These include the posterior communicating arteries (PCoA) and anterior communicating artery (ACoA) in the Circle of Willis, as well as the leptomeningeal collaterals which connect the large arterioles (LA) of the middle region to those of the anterior and posterior regions. Downstream of LA, compartments are defined for the small arterioles (SA), capillaries (CAP), small venules (SV), and large venules (LV). Blood flow is drained by the veins (V) and venous sinuses (VS). Compartments that are vasoactive are highlighted in yellow. Subscripts l and r correspond to left and right, and subscripts m,a, and p represent the middle, anterior, and posterior regions.

Figure 1 depicts the vascular network modeled in this study that is adapted from the cerebral vascular network in Ref. [27] to include capacitors, which model compliance, in the large arteries, veins, and venous sinuses. Such networks with components analogous to electrical circuits have been used to model portions of the cerebral circulation in previous studies [26, 28, 29]. Here, the arrangement and scaling of arteries are based on the general structure of this region in a human who has a complete Circle of Willis [30, 31]. The model includes the middle, posterior, and anterior regions on both the left and right sides (symmetric) of the brain. Blood flow entering the brain through the internal carotid artery (ICA) is supplied to the middle region by the MCA and to the anterior branch via the anterior cerebral artery (ACA). The basilar artery (BA) and posterior cerebral artery (PCA) primarily supply the posterior region, which is connected to the MCA and ACA by the posterior communicating artery (PCoA), a collateral artery within the Circle of Willis. The other collateral artery located within the Circle of Willis is the anterior communicating artery (ACoA), which joins the anterior regions of the left and right sides of the brain. The PCA is divided into the portion before the connection with the PCoA, part 1, and the portion after this connection, part 2. The ACA is also divided into pre‐communicating artery (ACA1) and post‐communicating artery (ACA2) portions. The microcirculation of the middle, posterior, and anterior regions is represented by compartments corresponding to the large arterioles (LA), small arterioles (SA), capillaries (CAP), small venules (SV), and large venules (LV). The two types of leptomeningeal collateral arteries most relevant to MCA occlusion are included; these collaterals connect the middle and anterior regions (COL_am_) and the middle and posterior regions (COL_pm_) and are considered part of the microcirculation regions. Blood is drained from the entire system by the veins (V) and venous sinuses (VS). The left and right sides of the cerebral vasculature contain the same types of vessels organized in the same configuration.

Each vessel type in the model is represented by a resistor. In the microcirculation regions, each resistor corresponds to multiple vessels of the same type arranged in parallel. Since larger vessels have increased elasticity and vascular compliance decreases in small arteries and arterioles [10], capacitors are included for arteries, veins, and venous sinuses, namely the following compartments: ICA, MCA, PCA1, PCA2, ACA1, ACA2, PCoA, ACoA, V, and VS. These compartments are divided into resistances *a* and *b* on either side of a capacitor (Figure 1, blue). Vessels in parts *a* and *b* are assumed to have the same diameter while each part is assumed to contain half of the total compartment resistance and length. To determine the pressures at the nodes where capacitors and resistors meet, a differential‐algebraic system of equations representing conservation of flow is solved (see Equation 1).

### 2.2 Hemodynamics

At pressure nodes with associated capacitors, the relationship between capacitance, pressure, and volume is used to equate the volume change to the net flow:

(1) $$\begin{array}{c} C_{\mathrm{BA}}\frac{d\left(P_{\mathrm{BA}1}-P_{\mathrm{ic}}\right)}{\mathrm{dt}}=\frac{P_{a}-P_{\mathrm{BA}1}}{R_{\mathrm{BAa}}}-\frac{P_{\mathrm{BA}1}-P_{\mathrm{BA}2}}{R_{\mathrm{BAb}}}\\ C_{\mathrm{ICA},l}\frac{d\left(P_{\mathrm{ICA},l1}-P_{\mathrm{ic}}\right)}{\mathrm{dt}}=\frac{P_{a}-P_{\mathrm{ICA},l1}}{R_{\mathrm{ICA},\mathrm{la}}}-\frac{P_{\mathrm{ICA},l1}-P_{\mathrm{ICA},l2}}{R_{\mathrm{ICA},\mathrm{lb}}}\\ \begin{array}{c} C_{\mathrm{MCA},l}\frac{d\left(P_{\mathrm{MCA},l}-P_{\mathrm{ic}}\right)}{\mathrm{dt}}=\frac{P_{\mathrm{ICA},l2}-P_{\mathrm{MCA},l}}{R_{\mathrm{MCA},\mathrm{la}}}-\frac{P_{\mathrm{MCA},l}-P_{1,\mathrm{ml}}}{R_{\mathrm{MCA},\mathrm{lb}}}\\ C_{\mathrm{ACA}1l}\frac{d\left(P_{\mathrm{ACA},l1}-P_{\mathrm{ic}}\right)}{\mathrm{dt}}=\frac{P_{\mathrm{ICA},l2}-P_{\mathrm{ACA},l1}}{R_{\mathrm{ACA}1\mathrm{la}}}-\frac{P_{\mathrm{ACA},l1}-P_{\mathrm{ACA},l}}{R_{\mathrm{ACA}1\mathrm{lb}}}\\ \begin{array}{c} C_{\mathrm{ACA}2l}\frac{d\left(P_{\mathrm{ACA},l2}-P_{\mathrm{ic}}\right)}{\mathrm{dt}}=\frac{P_{\mathrm{ACA},l}-P_{\mathrm{ACA},l2}}{R_{\mathrm{ACA}2\mathrm{la}}}-\frac{P_{\mathrm{ACA},l2}-P_{1,\mathrm{al}}}{R_{\mathrm{ACA}2\mathrm{lb}}}\\ C_{\mathrm{PCA}1l}\frac{d\left(P_{\mathrm{PCA},l1}-P_{\mathrm{ic}}\right)}{\mathrm{dt}}=\frac{P_{\mathrm{BA}2}-P_{\mathrm{PCA},l1}}{R_{\mathrm{PCA}1\mathrm{la}}}-\frac{P_{\mathrm{PCA},l1}-P_{\mathrm{PCA},l}}{R_{\mathrm{PCA}1\mathrm{lb}}}\\ \begin{array}{c} C_{\mathrm{PCA}2l}\frac{d\left(P_{\mathrm{PCA},l2}-P_{\mathrm{ic}}\right)}{\mathrm{dt}}=\frac{P_{\mathrm{PCA},l}-P_{\mathrm{PCA},l2}}{R_{\mathrm{PCA}2\mathrm{la}}}-\frac{P_{\mathrm{PCA},l2}-P_{1,\mathrm{pl}}}{R_{\mathrm{PCA}2\mathrm{lb}}}\\ C_{V}\frac{d\left(P_{6,\mathrm{mid}}-P_{\mathrm{ic}}\right)}{\mathrm{dt}}=\frac{P_{6}-P_{6,\mathrm{mid}}}{R_{\mathrm{Va}}}-\frac{P_{6,\mathrm{mid}}-P_{7}}{R_{\mathrm{Vb}}}\\ \begin{array}{c} C_{\mathrm{VS}}\frac{d\left(P_{7,\mathrm{mid}}-P_{\mathrm{ic}}\right)}{\mathrm{dt}}=\frac{P_{7}-P_{7,\mathrm{mid}}}{R_{\mathrm{VSa}}}-\frac{P_{7,\mathrm{mid}}-P_{V}}{R_{\mathrm{VSb}}}\\ \begin{array}{c} C_{\text{PCoA},l}\frac{d\left(P_{\text{PCoA},l}-P_{\mathrm{ic}}\right)}{\mathrm{dt}}=\frac{P_{\mathrm{PCA},l}-P_{\text{PCoA},l}}{R_{\text{PCoA},\mathrm{la}}}-\frac{P_{\text{PCoA},l}-P_{\mathrm{ICA},l2}}{R_{\text{PCoA},\mathrm{lb}}}\\ C_{\text{ACoA}}\frac{d\left(P_{\text{ACoA}}-P_{\mathrm{ic}}\right)}{\mathrm{dt}}=\frac{P_{\text{ACAr}}-P_{\text{ACoA}}}{R_{\text{ACoAa}}}-\frac{P_{\text{ACoA}}-P_{\text{ACAl}}}{R_{\text{ACoAb}}} \end{array} \end{array} \end{array} \end{array} \end{array} \end{array}$$

The pressures at the locations of capacitors on the right side of the Circle of Willis, $\begin{array}{c} P_{\text{ICA},r1},P_{\text{MCA},r},P_{\text{ACA},r1},P_{\text{ACA},r2},P_{\text{PCoA},r},P_{\text{PCA},r1}, \end{array}$ and $P_{\text{PCA},r2}$, are found using the same relationships applied at their corresponding left side locations in the equations above. The algebraic equations in the differential‐algebraic system are obtained by applying Kirchoff's Law at the nodes without capacitors, where intracranial pressure $P_{\mathrm{ic}}$ = 9.5 mmHg is assumed constant [26], C is the capacitance of the vessel compartment, and R is its resistance. Capacitance is calculated as the product of vessel distensibility and vessel volume. Distensibility reflects the elasticity and stiffness of vessels and is defined by the change in diameter in relation to change in blood pressure [32]. For arteries, the distensibility is estimated to be 5.35% per mmHg [6] and the distensibility of venous compartments is defined as 8 times the distensibility of the arteries [24]. Vessel volume is determined by lengths and diameters. Resistance ($R_{i}$) is also determined by vessel length ($L_{i}$) and diameter ($D_{i}$) using Poiseuille's Law: $R_{i}=\frac{128L_{i}\mu }{n_{i}\pi D_{i}^{4}}$, where $\;n_{i}$ is the number of vessels contained in compartment i and μ is the viscosity of blood with constant value 0.04 Poise [26].

**FIGURE 2:**
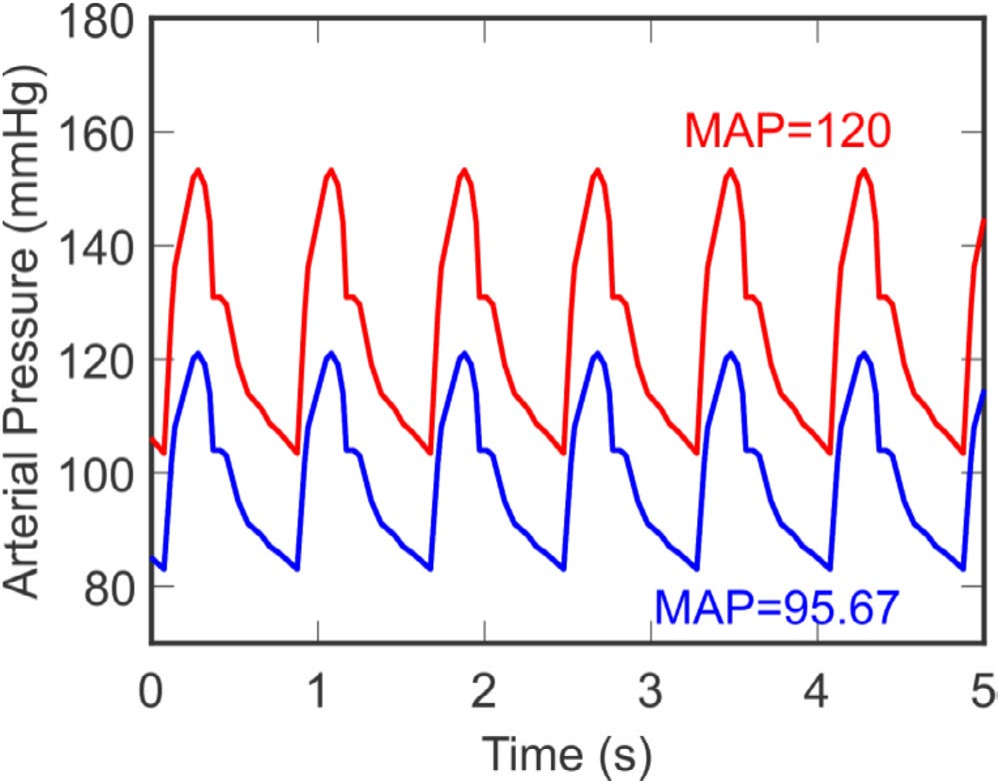
Time‐dependent arterial pressure boundary condition. Arterial pressure as a function of time ($P_{a}\left(t\right)$) is used as the incoming boundary condition and was obtained using the measured carotid pressure waveform from Haluska et al. [33]. Incoming pressure $P_{a}\left(t\right)$ corresponds to the control mean arterial pressure of 95.67 mmHg (blue) and an elevated MAP of 120 mmHg (red). An MAP of 120 mmHg is obtained by a combination of shifting $P_{a}\left(t\right)$ and increasing its amplitude.

A pulsatile boundary condition is used for incoming arterial pressure ($P_{a}$) to account for variations in blood flow entering the system due to the cardiac cycle. The condition is based on measurements of the aortic pressure wave from Haluska et al. [33]. Linear interpolation between points on the measured pressure wave is used to construct the periodic waveform for arterial pressure shown by the blue curve in Figure 2. This expression for $P_{a}\left(t\right)$, which corresponds to a mean arterial pressure of 95.67 mmHg, is used as the inlet condition for the system in the control state (see Section 2.4).

### 2.3 Flow Regulation

The diameters of the arterioles and leptomeningeal collaterals are regulated by changes in smooth muscle activation that depend on transmural pressure, shear stress, and metabolic demand. These vasoactive compartments are highlighted in Figure 1. The remaining compartments are treated as fixed resistances. In the large and small arterioles and downstream collaterals, changes in diameters ($D_{i}$) and activations ($A_{i}$) are given by the equations

(2) $$\frac{dD_{i}}{\mathrm{dt}}=\frac{1}{\tau_{d}}\frac{D_{c,i}}{T_{c,i}}\left(T_{i}-T_{\text{total},i}\right)$$

$$\frac{dA_{i}}{\mathrm{dt}}=\frac{1}{\tau_{a}}\left(A_{\text{total},i}-A_{i}\right)$$

where $\tau_{d}=1\;s$ and $\tau_{a}=60\;s$ are diameter and activation time constants, $D_{c,i}$ and $T_{c,i}$ are the control values of diameter and tension, respectively (see Section 2.4), and index $i=LA_{\mathrm{kj}},SA_{\mathrm{kj}},\mathrm{CO}L_{\mathrm{amj}},\mathrm{CO}L_{\mathrm{pmj}}$ indicates the vessel type, with subscript k=m,a,p for the middle, anterior, or posterior regions and j=l,r for the left or right sides of the brain. As described in Ref. [34], the time constants $\tau_{d}$ and $\tau_{a}$ are chosen based on the time taken for the diameter of arterioles to reach a new equilibrium after an incremental increase in arterial pressure and govern the rate at which the equations reach their steady state values.

*COL*_*amj*_ and *COL*_*pmj*_ denote leptomeningeal collaterals connecting the anterior to middle regions (amj subscript) or posterior to middle regions (pmj subscript) on either the left or right side of the brain. Circumferential wall tension is given by the Law of Laplace as $T_{i}=\frac{\left({\bar{P}}_{i}-P_{\mathrm{ic}}\right)D_{i}}{2}$ where the average pressure drop across the vessel wall is equal to the difference between the average intravascular pressure ${\bar{P}}_{i}$, and the surrounding intracranial pressure $P_{\mathrm{ic}}$. The average intravascular pressure is found by averaging the solutions of Equation (1) over a time interval of one period, where the period of the solutions is the same as that of the incoming arterial pressure $P_{a}\left(t\right)$. Total tension ($T_{\text{total},i}$) in a compartment is the sum of passive and active tension as defined by Carlson et al. [35, 36] and Arciero et al. [34]; the parameters in the passive and active tension functions are estimated in Ref. [27] for cerebral arterioles.

Diameters of the arterioles are regulated by the degree of smooth muscle tone, which is represented by activation (A). Total activation is a sigmoidal function of a stimulus $S_{\text{tone}}$ that depends on the local hemodynamic conditions: $A_{\text{total}}=\frac{1}{1+\exp\left(-S_{\text{tone}}\right)}$. The stimulus for smooth muscle activation is given by

(3) $$S_{\text{tone},i}=\left\{\begin{array}{c} C_{\mathrm{myo},i}T_{i}-C_{\text{shear},i}\tau_{i}-C_{\text{meta},i}S_{\text{meta},i}+C_{\text{tone},i}^{\prime \prime },i=\mathrm{SA},\mathrm{COL}\\ C_{\mathrm{myo},i}T_{i}-C_{\text{shear},i}\tau_{i}+C_{\text{tone},i}^{\prime \prime },i=\mathrm{LA} \end{array}\right.$$

where the myogenic response is based on circumferential wall tension ($T_{i}$) calculated from transmural pressure and vessel diameter. The shear response depends on wall shear stress $\tau_{i}=\frac{32\mu Q_{i}}{\pi D_{i}^{3}}$, where $Q_{i}$ is vessel blood flow calculated using the average values of pressure at each node. $S_{\text{meta},i}$ is the conducted response signal generated in the venules and conducted upstream to the small arterioles and collaterals. Details on the signal calculation are given in Zhao et al. [27].

### 2.4 Control State

Before allowing for autoregulation and changes of resistance in the large arteriole (LA), small arteriole (SA), and collateral (COL) compartments, certain quantities and properties are determined in a control (reference) state. Diameter and length of the arteries in the Circle of Willis, which includes the ICA, BA, MCA, ACA1, ACA2, PCA1, PCA2, PCoA, and ACoA, are assumed to have the same values as in the model from Ref. [27]. These values are listed in Table 2. Parts *a* and *b* of an artery have the same diameters while each part contains half of the artery's length, and thus accounts for half of its total resistance. Compartments corresponding to arteries each contain one vessel. The lengths of the large and small arterioles in all microcirculation regions are set to their values from Ref. [27], where the lengths of the LV and SV follow from assumed symmetry between the arterioles and venules. Values from Ref. [27] are also used to set the length and resistance of the V and VS, the shear stress of the LA, SA, CAP, SV, and LV, and the number of vessels (temporarily) in the LA, SA, CAP, SV, and LV compartments. The length and resistance of the V and VS can be used to obtain the diameters. Length, diameter, and number are used to calculate resistance for the arteries in the Circle of Willis and for the capillary compartments. Capacitance of arteries and veins is found using volumes calculated from control diameter and distensibility. The distensibility of arteries is $4.01\times 10^{-7}$ cm^2^/dyn based on measurements of MCA distensibility from Ref. [37]. Distensibility of veins is assumed to be eight times the arterial distensibility according to Ref. [24].

The diameter differential equations for the LA, SA, and COL (Equation 2) of each region are then solved with a fixed activation of 0.5 in the LA and SA and 0.99 in the COL, as in Refs. [27, 38]. Within each time step of solving these differential equations, an iterative procedure is invoked which uses calculations of resistance, pressure, and flow to update the numbers of vessels in each microcirculation compartment and terminates when these numbers converge to a fixed value.

More specifically, the diameters of the SV and LV are first obtained by using the current SA and LA diameters and symmetry conditions. Then, resistance of the microcirculation compartments is updated using Poiseuille's Law. The differential and linear equations for pressures in Equation (1) are solved on a short time interval; time averaged values of the pressure at each node are determined by averaging the pressure versus time function over the last period, $\bar{P_{i}}=\frac{1}{T}\int_{t_{\mathrm{end}-T}}^{t_{\mathrm{end}}}P_{i}\left(t\right)\mathrm{dt}$, where $t_{\mathrm{end}}$ is the final time point in the solution and the period of averaging T is the period of the $P_{a}\left(t\right)$ boundary condition. Flow through compartments in the microcirculation are found using Ohm's Law and the averaged nodal pressures; flow through individual vessels ($Q_{i}$) are calculated as $Q_{i}=\frac{\pi \tau_{i}D_{i}^{3}}{32\mu }$. Next, the number of vessels in the LA, SA, CAP, SV, and LV compartments is updated by dividing compartment flow by individual vessel flow. These steps are repeated until the vessel numbers converge.

The time‐dependent averages are used as the control pressures at the nodes. Control pressures of the LA, SA, and COL are found by taking the midpoint of the averaged values:

(4) $$P_{c,\text{LAij}}=\frac{\bar{P_{1\mathrm{ij}}}+\bar{P_{2\mathrm{ij}}}}{2};P_{c,\text{SAij}}=\frac{\bar{P_{2\mathrm{ij}}}+\bar{P_{3\mathrm{ij}}}}{2};P_{c,\text{COLimj}}=\frac{\bar{P_{2\mathrm{ij}}}+\bar{P_{2\mathrm{mj}}}}{2}$$

where j=l,r for the left and right sides of the brain, i=m,a,p in the LA and SA for the middle, anterior, and posterior regions, and i=p,a in the COL for collaterals connecting the middle to posterior or middle to anterior regions. A complete list of control pressure values is given in Table 1, where average refers to the integral average taken over one period of the solution. Other control state quantities are provided in Table 2.

**TABLE 1:** Control state pressure values.

Node	Average pressure (mmHg)
$P_{a}$	99.2
$P_{\mathrm{ICA},j1}$	97.9
$P_{\mathrm{ICA},j2}$	96.6
$P_{\text{MCAj}}$	94.2
$P_{\text{ACAj}1}$	94.6
$P_{\text{ACAj}}$	92.5
$P_{\text{ACAj}2}$	91.6
$P_{\mathrm{BA}1}$	98.6
$P_{\mathrm{BA}2}$	97.9
$P_{\text{PCAj}1}$	97.4
$P_{\text{PCAj}}$	96.8
$P_{\text{PCAj}2}$	94.3
$P_{\text{PCoAj}}$	96.7
$P_{\text{ACoA}}$	92.5
$P_{1,\mathrm{mj}}$	91.7
$P_{2,\mathrm{mj}}$	74.7
$P_{3,\mathrm{mj}}$	44.7
$P_{4,\mathrm{mj}}$	16.0
$P_{5,\mathrm{mj}}$	14.5
$P_{1,\mathrm{aj}}$	90.8
$P_{2,\mathrm{aj}}$	74.6
$P_{3,\mathrm{aj}}$	44.7
$P_{4,\mathrm{aj}}$	15.9
$P_{5,\mathrm{aj}}$	14.5
$P_{1,\mathrm{pj}}$	91.8
$P_{2,\mathrm{pj}}$	74.7
$P_{3,\mathrm{pj}}$	44.7
$P_{4,\mathrm{pj}}$	16.0
$P_{5,\mathrm{pj}}$	14.5
$P_{6}$	13.7
$P_{6,\mathrm{mid}}$	12.7
$P_{7}$	11.6
$P_{7,\mathrm{mid}}$	10.6
$P_{V}$	9.5

*Note:* *j* = *l*, *r* for left or right.

**TABLE 2:** Control state vessel diameter, length, resistance, number, and capacitance values.

Vessel	Diameter (μm)	Length (cm)	Resistance (dyn s/cm^5^)	Number	Capacitance (cm^5^/dyn)
$\mathrm{IC}A_{j}$	4100	13.15	758	1	6.9680 × 10^−7^
BA	3400	4.92	600	1	1.7928 × 10^−7^
$\mathrm{MC}A_{j}$	2800	7.25	1922	1	1.7917 × 10^−7^
$\mathrm{PCA}1_{j}$	2000	1	1019	1	1.2609 × 10^−8^
$\mathrm{PCA}2_{j}$	2000	4.72	4808	1	5.9514 × 10^−8^
$\mathrm{ACA}1_{j}$	1500	1.57	5054	1	1.1135 × 10^−8^
$\mathrm{ACA}2_{j}$	1500	0.672	2163	1	4.7661 × 10^−9^
$\mathrm{PCo}A_{j}$	720	2	1213	1	3.2682 × 10^−9^
ACoA	800	0.5	1989	1	1.0087 × 10^−9^
$LA_{\mathrm{mj}}$	681	7.19	6784	80	—
$SA_{\mathrm{mj}}$	148	2.76	11 943	7746	—
$\mathrm{CA}P_{\mathrm{mj}}$	6.47	0.115	11 421	9.3646 × 10^7^	—
$SV_{\mathrm{mj}}$	319	2.76	561	7746	—
$LV_{\mathrm{mj}}$	1463	7.19	319	80	—
$LA_{\mathrm{aj}}$	679	6.79	20 236	26	—
$SA_{\mathrm{aj}}$	148	2.76	37 618	2464	—
$\mathrm{CA}P_{\mathrm{aj}}$	6.47	0.115	35 957	2.9745 × 10^7^	—
$SV_{\mathrm{aj}}$	319	2.76	1766	2464	—
$LV_{\mathrm{aj}}$	1459	6.79	950	26	—
$LA_{\mathrm{pj}}$	681	7.21	16 473	33	—
$SA_{\mathrm{pj}}$	148	2.76	28 923	3198	—
$\mathrm{CA}P_{\mathrm{pj}}$	6.47	0.115	27 660	3.8668 × 10^7^	—
$SV_{\mathrm{pj}}$	319	2.76	1358	3198	—
$LV_{\mathrm{pj}}$	1463	7.21	774	33	—
$\mathrm{CO}L_{\mathrm{amj}}$	317	0.4379	140 480	5	—
$\mathrm{CO}L_{\mathrm{pmj}}$	354	0.6213	160 610	4	—
V	5553	7	240	1	5.4428 × 10^−6^
VS	6071	10	240	1	9.2934 × 10^−6^

*Note:* *j* = *l*, *r* for left or right. Parts a and b of arteries have the same diameter and number of vessels, and each part contains half of the length and resistance.

### 2.5 Varying Arterial Pressure

To examine the influence of capacitors on flow autoregulation, the incoming pressure ($P_{a}$) is changed by varying mean arterial pressure (MAP) between 40 and 150 mmHg. Each variation in mean arterial pressure corresponds to changes in the waveform representing the incoming pressure from the carotid artery. The shifting and rescaling of the waveform for different mean arterial pressures is based on measurements from Gavish et al. [39], which monitored blood pressures in 140 subjects over a period of 24 h. In general, they found an approximately linear relationship between systolic and diastolic pressure, with data suggesting this linear relationship could involve a nonzero intercept. This observation indicates that pulse pressure is not necessarily proportional to MAP. To vary pressures more realistically (i.e., instead of rescaling $P_{a}\left(t\right)$ by a constant), we implement the following procedure.

We first obtain an approximate linear function that relates systolic (S) and diastolic (Dy) pressures. The vertical intercept of the line is obtained by assuming that its slope is 1.58, taken from the first figure in Gavish et al. [39], and that the maximum (control systolic pressure, $S_{c}$) and minimum (control diastolic pressure, ${\mathrm{Dy}}_{c}$) of the original pressure wave in the control state constitutes a point on the line. This eventually yields: S=1.58Dy−10.14.

Next, this relationship is used to rescale and shift the original (control) pressure wave. For each point on the original pressure wave, a new point corresponding to the varied (non‐control) value of MAP is found using the relationship $P_{\mathrm{new}}\left(t\right)=a_{i}P_{\text{cont}}\left(t\right)+b_{i}$, where $P_{\mathrm{new}}\left(t\right)$ and $P_{\text{cont}}\left(t\right)$ are points on the shifted and control pressure wave, respectively. The definition of MAP in terms of systolic (S) and diastolic (Dy) pressure, $\mathrm{MAP}=\mathrm{Dy}+\frac{1}{3}\left(S-\mathrm{Dy}\right)$, can be combined with S=1.58Dy−10.14 to obtain expressions for systolic and diastolic pressures in terms of MAP. These expressions are related to the control values of systolic and diastolic pressure ($S_{c}$ and ${\mathrm{Dy}}_{c}$) by $S=a_{i}S_{c}+b_{i}$ and $\mathrm{Dy}=a_{i}{\mathrm{Dy}}_{c}+b_{i}$, where S=1.324MAP−5.664, $\mathrm{Dy}=\frac{\mathrm{MAP}+3.38}{1.193}$, $S_{c}=121$ mmHg, and ${\mathrm{Dy}}_{c}=83$ mmHg. The values of $a_{i}$ and $b_{i}$ in terms of MAP are determined and take on different values for each MAP investigated. Thus, the rescaling and vertical shift of the pressure function is unique to the given value of MAP.

Figure 2 shows the incoming pressure wave boundary condition $P_{a}\left(t\right)$ for two different values of mean arterial pressure. The blue curve corresponds to the control value of 95.67 mmHg, and the red curve depicts an elevated mean arterial pressure of 120 mmHg [5]. The changes in the red pressure wave constitute both a vertical shift and a rescaling of the function. Zheng et al. [5] associated both MAP and pulse pressure with ischemic stroke in individuals who have uncontrolled hypertension, and thus the modeled changes in the pressure wave simulate an elevation in diastolic, systolic, and mean arterial pressure as well as an increased amplitude corresponding to a larger pulse pressure. This transformation of $P_{a}\left(t\right)$ facilitates the analysis of how changes in both mean arterial pressure and pulse pressure affect blood flow compensation following MCA occlusion.

### 2.6 Model Simulations

**FIGURE 3:**
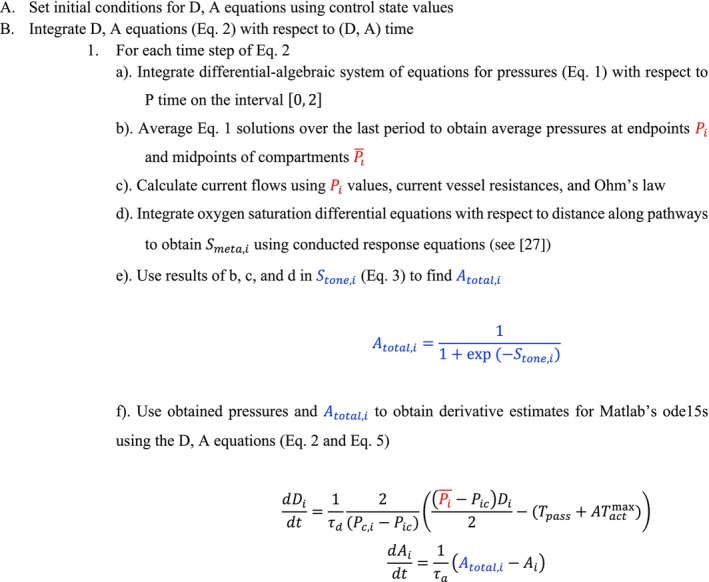
Algorithm used for solving the diameter and activation differential equations in numerical simulations.

Outside of the control state, the equations for diameter and activation in the LA, SA, and COL vessels of each region can be solved for different values of oxygen demand or mean arterial pressure in the presence or absence of an occlusion of the left MCA. The change in diameters is given by

(5) $$\begin{array}{c} \begin{array}{c} \frac{dD_{i}}{\mathrm{dt}}=\frac{1}{\tau_{d}}\frac{2}{\left(P_{c,i}-P_{\mathrm{ic}}\right)}\\ \left(\frac{\left(\bar{P_{i}}\left(D_{i},A_{i}\right)-P_{\mathrm{ic}}\right)D_{i}}{2}-\left(T_{\text{pass}}\left(D_{i},A_{i}\right)+A_{i}T_{\mathrm{act}}^{\max}\left(D_{i},A_{i}\right)\right)\right) \end{array} \end{array}$$

where *i* = LA, SA, or COL and $\bar{P_{i}}$ denotes the time averaged value of pressure at the midpoint of the compartment. Since changes in diameter affect resistance, the pressure differential and linear equations (Equation (1)) are solved using updated resistances on a short time interval [0,2] (“P Time”). The pressure versus time solutions of these equations $P_{i}\left(t\right)$ are averaged over the last period to obtain $\bar{P_{i}}$. The process of finding and averaging solutions to Equation (1) is repeated at every discrete time step of solving the diameter and activation differential equations (“D, A Time”) to obtain updates in average pressures corresponding to diameter values at the given time step. An illustration of this procedure is shown in Figure 3. When resistances are significantly changed, the pressure solutions may be expected to take some time to converge to new physiologically realistic quasi‐steady periodic states. However, since every discrete time step is small, resistance changes are also small, leading to quick convergence (Figure 5A).

To simulate total occlusion of the left MCA, the diameter of parts *a* and *b* of the left MCA is set to zero. Since there is no flow to the middle left large arterioles in this situation, the changes in diameter and activation of this compartment are no longer tracked. Furthermore, the differential‐algebraic system of equations solving for the pressures is rewritten to exclude the variables $P_{\mathrm{MCA},l}$ and $P_{1,\mathrm{ml}}$.

## 3 Results

**FIGURE 4:**
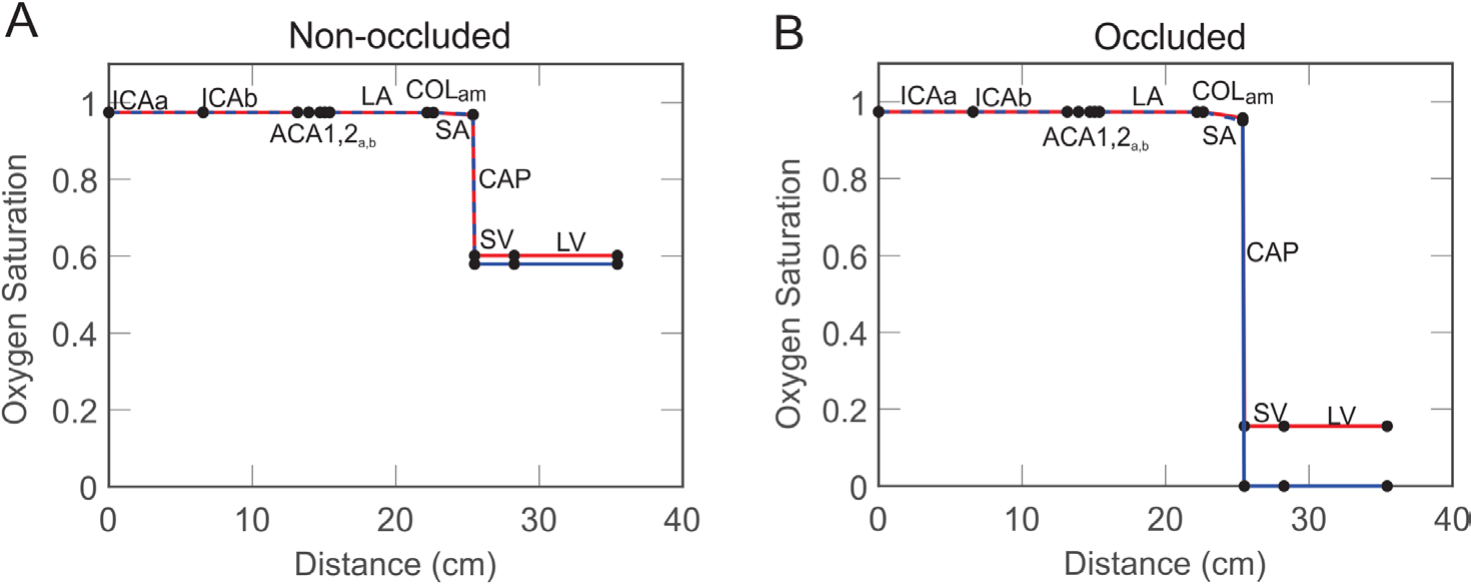
Oxygen saturation along the pathway which connects the arteries and LA of the anterior left branch to the SA, CAP, SV, and LV of the middle left through the anterior‐middle collaterals. (A) Non‐occluded. (B) Occluded. Blue lines correspond to the control MAP and red lines correspond to an MAP of 120 mmHg.

Figure 4 shows the oxygen saturation along the pathway connecting the anterior to middle branches on the left side of the brain. This pathway consists of the arteries of the anterior left region (ICA_a_, ICA_b_, ACA1_a_, ACA1_b_, ACA2_a_, ACA2_b_, and LA_a_), the collaterals connecting the anterior large arterioles to the middle branch, and the remaining microcirculation of the middle branch (SA_m_, CAP_m_, SV_m_, and LV_m_). In the occluded case, the oxygen levels at the end of the middle branch provide an indication to the extent of flow compensation by the collaterals. These levels are compared to the non‐occluded case and the deficit in oxygen saturation is discussed in detail in previous work [27]. To analyze how an elevated arterial pressure affects flow restoration, the incoming pressure function is transformed to correspond to an MAP of 120 mmHg. Figure 4A depicts the non‐occluded case and Figure 4B the occluded case, where red lines correspond to MAP = 120 mmHg and blue lines correspond to the control MAP value. For a moderately high value of oxygen demand ($M_{0}=12$ cm^3^ O_2_/100 cm^3^/min), the elevation of incoming arterial pressure prevents the venous oxygen saturation at the end of the middle‐left pathway from approaching zero when the left MCA is occluded. Therefore, the deficit in oxygen saturation between the occluded and non‐occluded cases is reduced by the increased pressure, indicating improved flow compensation. In the absence of occlusion, the increase in MAP does not have a significant impact on venous oxygen saturation.

**FIGURE 5:**
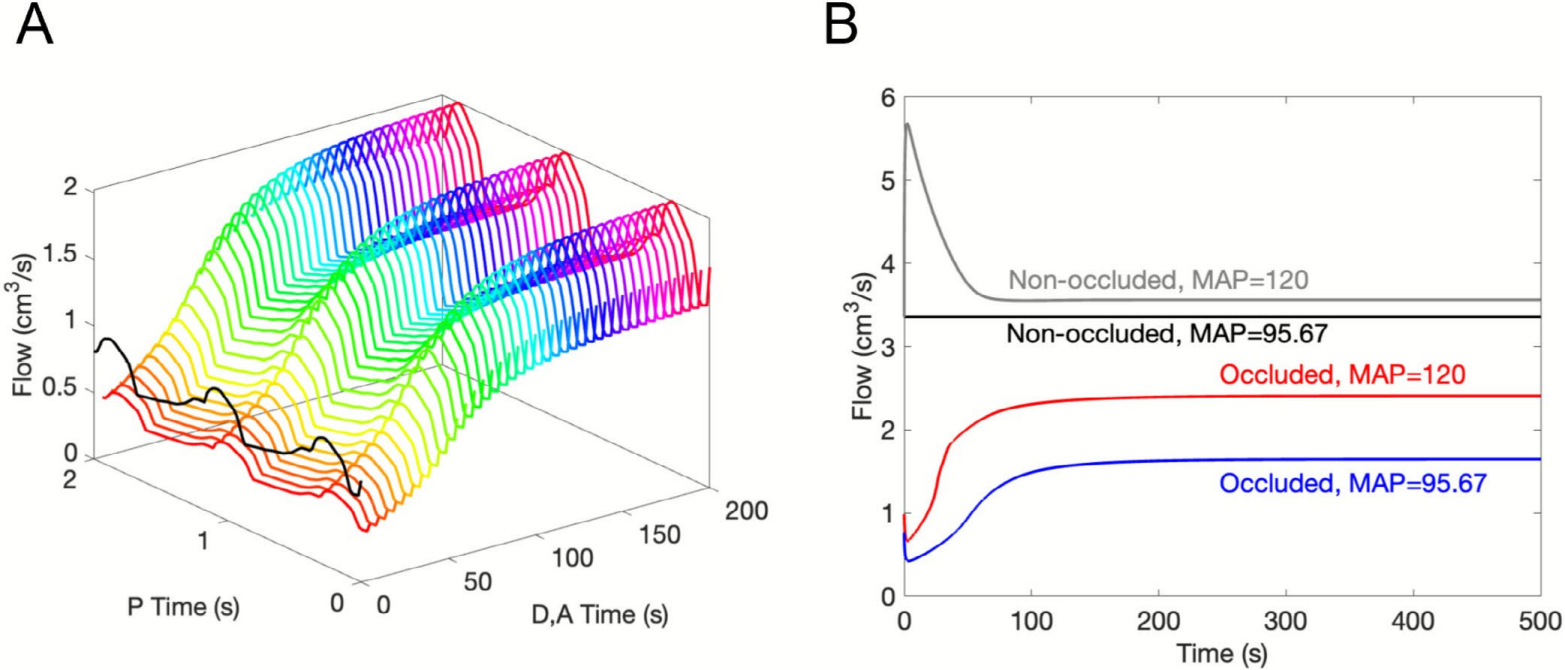
(A) Dependence of blood flow on both diameter‐activation and pressure equations. Flow to the middle‐left region in the occluded case is shown as a function of the time intervals for the diameter‐activation (axis labeled D,A Time) and pressure (P Time) differential equations. Changes in flow from 0 to 2 s (P Time) at each point in time of the diameter‐activation equations are shown. (B) Comparison of changes in total blood flow to the middle‐left region after occlusion (blue and red) relative to the non‐occluded levels (black and gray). The blue and black curves correspond to normal MAP (95.67 mmHg); red and gray represent both elevated MAP (120 mmHg) and pulse pressure.

Changes in total blood flow to the middle‐left region following MCA occlusion are shown in Figure 5. Flow to a region consists of the total flow through the small arteriole compartment and thereby also through the capillaries and venules. Figure 5A illustrates the relationship between the pressure differential equations (Equation (1)) and the diameter and activation equations (Equation (2)). Panel A combines the time scales of both Equations (1) and (2) by demonstrating the dependence of blood flow on each time variable. The axis labeled “D,A Time” corresponds to time points in the solutions of the equations for diameters and activations in the vasoactive compartments. The “P Time” axis corresponds to the time interval for solutions of Equation (1), and the curves parallel to the P Time and Flow axes show the changes in blood flow over these 2 s intervals. Moving along the D,A Time direction shows increases in blood flow over the interval from 0 to 200 s that correspond to solutions of the diameter and activation equations. The values of flow used in the responses that determine activation are the averages over the last period of the curves parallel to the P Time–Flow plane.

The blue curve in Figure 5B shows only these averages versus their corresponding times in D,A time/normal time for the control MAP value. These values are compared to post‐occlusion flow in the middle‐left when MAP and pulse pressure are increased to correspond to MAP = 120 mmHg (red curve), non‐occluded flow in the control case (black), and non‐occluded flow with MAP = 120 mmHg (gray). Although the level of flow immediately following occlusion is very low, flow recovers somewhat over time due to the dilation and redirection of flow through the leptomeningeal collaterals. The magnitude of flow recovery is enhanced with the elevation of MAP, but the time at which flow attains its maximum remains unchanged. In all cases, occluded flow is never able to recover to non‐occluded levels. Similar to the trend in oxygen saturation displayed in Figure 4, in the non‐occluded case, the higher MAP has a minimal effect on the steady state level of blood flow, increasing it by only 6% (Figure 5B, black and gray curves). However, in the presence of occlusion, the increase in MAP from 95.67 to 120 mmHg results in a 46% increase in blood flow (Figure 5B, red and blue curves). This observation suggests that when the MAP is elevated, there is not only an increase in total flow to the system, but also a possible redistribution of flow so that a larger portion is redirected to the middle region through collateral vessels.

**FIGURE 6:**
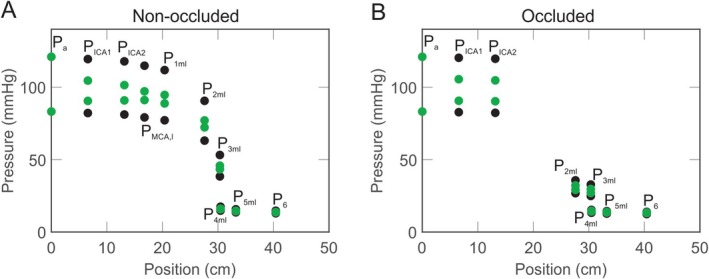
Effects of distensibility on the amplitude of oscillations at pressure nodes. Maximum and minimum values of the time‐dependent pressures along the middle‐left pathway under non‐occluded (panel A) and occluded (panel B) conditions. $P_{\text{MCA,l}}$ and $P_{1,\mathrm{ml}}$ are eliminated from B since these pressures are not calculated in the occluded case. Black points correspond to using the control value of distensibility of the arteries and veins, whereas green points are obtained with distensibility increased by a factor of 1000.

Figure 6 examines time‐dependent pressure values given by the solutions of Equation (1). The maximum and minimum values of the pressure solutions over one period of the cardiac cycle for the pressure nodes along the middle‐left branch in the non‐occluded (A) and occluded cases (B) are shown. Black points correspond to simulations using the values of distensibility set in the control state, and green points are obtained using highly increased values of distensibility (1000×). At the downstream pressure nodes, the points are more condensed since the maxima and minima are closer when the oscillations in pressure are dampened by the capacitances of the upstream arteries. With increased distensibility, the arteries retain more flow in order to regularize pressure and flow patterns, which leads to reduced amplitudes of the time‐dependent pressures and further dampening of oscillations along the vascular pathway.

Furthermore, Figure 6 shows that following occlusion, the pressure at the end of the middle left small arterioles compartment ($P_{2,\mathrm{ml}}$) is significantly impacted by the lack of flow supply from the MCA. At this location, the average pressure or approximate midpoint of the oscillations is reduced by 59% under occluded conditions. This change is consistent between both the higher (green points) and lower (black points) values of distensibility, which indicates that variations in vessel distensibility have minimal impact on the reduction of the pressure perfusing the microcirculation following occlusion. A decrease in the amplitude of oscillations at $P_{2,\mathrm{ml}}$ occurs as expected due to the much lower average pressure. However, the magnitude of this effect appears to depend on distensibility. At the control value of distensibility, the amplitude of the pressure time dynamics decreased by 68% after occlusion, but at the high distensibility value, the amplitude only decreased by 39%. These observations suggest that while increased distensibility may not affect the pressure and flow perfusing the MCA‐dependent microcirculation, it could affect other factors associated with occlusion such as changes in the patterns of pressure oscillations.

**FIGURE 7:**
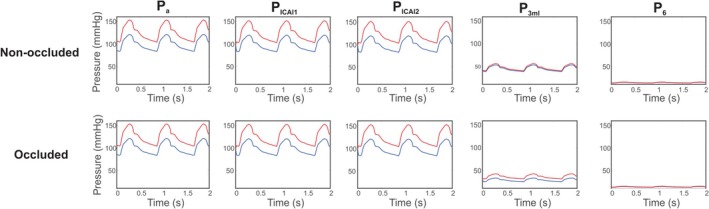
Time dynamics of pressures at locations along the middle‐left branch in the non‐occluded (top row) and occluded (bottom row) cases. MAP is increased from the control value (blue) to 120 mmHg (red).

The pressure waves corresponding to the control values of diameters and activations are shown at various points along the middle‐left branch in Figure 7. The top row corresponds to the non‐occluded case, and the bottom row shows pressures following MCA occlusion. The figure compares the control value of MAP (blue curves) with an elevated value of 120 mmHg (red curves). As the amplitude of oscillations is decreased at the downstream pressure nodes, the effects of the higher MAP are also reduced since differences between the two waves are lessened at nodes $P_{3,\mathrm{ml}}$ and $P_{6}$. There is a more pronounced difference between the pressure waves at $P_{3,\mathrm{ml}}$ in the occluded case than in the non‐occluded case, although the amplitude of the waves and the average pressure values at this location are reduced with occlusion.

**FIGURE 8:**
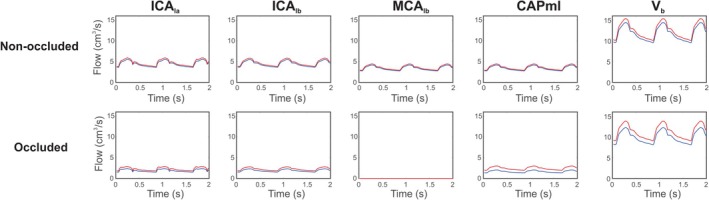
Time‐dependent blood flows in different vessels along the middle‐left pathway. Time dynamics in various compartments are shown using the control MAP (blue) and an elevated MAP of 120 mmHg (red). The same compartments are compared for both the non‐occluded (top row) and occluded (bottom row) cases.

Figure 8 shows the time dynamics of blood flow corresponding to the pressure dynamics in Figure 7. Flow results portray select compartments of the middle‐left branch for the control (blue) and elevated (red) values of MAP under non‐occluded (top row) and occluded (bottom row) conditions. In the bottom row, flow in part b of the MCA is zero since total occlusion is assumed. The levels of blood flow and the magnitude of the oscillations are both reduced in the occluded case. Similar to the trend observed in Figure 7 at $P_{3,\mathrm{ml}}$, the waves corresponding to blood flow at the two different arterial pressures are more separated from one another in the occluded case.

**FIGURE 9:**
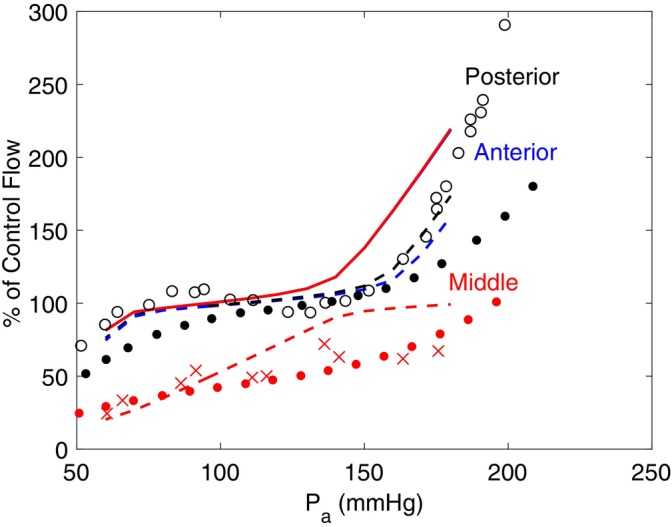
Autoregulation curves representing blood flow relative to changes in MAP for the middle (red), posterior (black), and anterior (blue) regions on the left side of the brain. Solid lines correspond to the non‐occluded case and dashed lines are with occlusion. Flow values shown are percentages of flow in the control state. Data points correspond to flow measurements from experiments taken in either healthy (black) or ischemic (red) conditions: • Dirnagl and Pulsinelli [40], o Hernandez et al. [41], × Symon et al. [42].

Autoregulation in the middle, anterior, and posterior regions of the left brain is examined in Figure 9, where solid curves correspond to the non‐occluded case and the dashed curve represents the occluded case. The values of blood flow shown for each MAP are the averages over one period of the cardiac cycle once steady state diameters have been reached and are shown as a percentage of total flow in the corresponding region in the control state. The autoregulation curves are compared with experimental data from multiple studies [40, 41, 42]. In healthy individuals, blood flow in the middle is consistently higher since the MCA and its downstream vasculature supplies more areas of the brain. Consistent with previous studies of cerebral autoregulation in Ref. [27], autoregulation of the middle region is disrupted by MCA occlusion. For pressures between 60 and 150 mmHg, where flow is maintained at a relatively constant level in the normal case, flow in the region of MCA occlusion increases approximately linearly with a slope of 0.95/mmHg. Similarly, Symon et al. observed a linear relationship between pressure and flow in regions of the brain affected by MCA occlusion, where the slope varied between 0.78/mmHg and 1.48/mmHg depending on the degree of ischemia. For a moderate degree of ischemia, the measured slope of 0.98/mmHg was in close agreement with model predictions [42]. When pressures above this range are considered, however, it appears that autoregulation is not lost, but rather the plateau in blood flow is shifted to higher values of MAP, which is consistent with prior results [27]. The experiment in Ref. [42] observed that when post‐occlusion blood flow exceeded 40% of the non‐occluded level, autoregulation is partially preserved, meaning that for values of pressure higher than the standard MAP, there exists a region of relatively constant slope on the autoregulation curve. However, there is no such region observed for pressures below the standard value of MAP. This observation suggests that the plateau may indeed be shifted to a higher range of pressures, and that the amount of this shift or the location of the new autoregulation plateau may depend on the degree of ischemia. As expected, the anterior and posterior regions are not significantly affected by the occlusion except at high values of MAP, where flow is lower than in the non‐occluded case.

## 4 Discussion

The present study examines the effects of a time‐dependent arterial pressure boundary condition on a model of the cerebral vasculature which includes MCA occlusion and the recruitment of leptomeningeal collateral vessels. This model is developed from integrating elements of capacitance and pressure dynamics into a preexisting model of cerebral blood flow regulation [27]. Incorporating the additional time dependency facilitates the study of pressure and flow patterns at various positions throughout the vascular network to reveal how oscillatory dynamics change between the arteries and the microcirculation, details that cannot be discerned from the previous model focused on steady‐state values. Variations in pressure pulsatility, as well as changes in blood flow and oxygen levels, both influence local blood flow regulation. Therefore, combining all of these aspects in a model of flow regulation is of great importance, particularly since disturbances in pulsatility from arterial stiffening, elevated arterial pressure, and deficits in blood flow due to occlusion have a high likelihood of occurring simultaneously. The investigation analyzes how a complete occlusion of the MCA affects the microcirculation of dependent tissue regions, with an emphasis on how variations in arterial pressure and vessel distensibility can alter compensatory responses and blood flow restoration following occlusion.

The distensibility of larger vessels and their ability to accommodate pulsatile flow resulting from the boundary condition is represented by capacitance elements. Mathematically, these elements involve relating volume changes to net flow at the location of each capacitor. Figure 5A provides a visual representation of how this component is combined with the equations for vessel diameter and activation to develop a model incorporating both cerebral blood flow regulation and pressure pulsatility. The figure shows the time dynamics of flow at multiple time points in the solution of the diameter and activation equations. As the level of blood flow changes on the longer time interval (axis labeled “D,A Time”), the solutions for flow on the interval 0–2 s (“P Time” axis) are vertically shifted, while maintaining the same period and overall shape. This trend indicates that changes in the patterns of the pressure and flow time dynamics on each 2 s interval do not significantly influence changes in blood flow levels on the long‐term time scale. Therefore, the averaging of the pressure equation solutions over the last period is a reasonable approach to determining the pressure and flow‐mediated responses of vascular tone.

Since there is a high degree of co‐occurrence between stroke and hypertension [43], the incoming boundary condition is varied to correspond to a higher mean arterial pressure. Hypertension is the most prevalent risk factor for stroke, and there is evidence to suggest that lowering blood pressure closer to the normal range can reduce this risk [16]. Figures 4 and 5B suggest that an elevated arterial pressure may improve stroke outcomes because the higher MAP enhances flow compensation to occluded regions by 46% and improves venous oxygen saturation from 0 to 0.16. However, the management of blood pressure in stroke cases is complex due to its varying effects on cerebral hemodynamics [16] as well as the need to distinguish among different types of hypertension. Although acute hypertension can potentially maintain better perfusion and oxygen levels, untreated chronic hypertension often induces remodeling of blood vessels such as thickening of the vessel wall which can hinder blood flow and oxygen transport to the ischemic area [44].

Since elevations in MAP alone or together with pulse pressure are associated with stroke in different demographics [5], the impact of either increasing both quantities or only MAP is assessed. Because there is very little difference between these two cases, the effects of increasing MAP with constant pulse pressure are not shown in Figure 5B, but it is possible that the effects of pulse pressure are minimized by using average pressures in model flow calculations. Zheng et al. [5] found a positive correlation between MAP and pulse pressure in individuals with hypertension, indicating that both quantities are likely to be elevated in hypertensive subjects, not just MAP. Furthermore, the finding that only MAP is associated with stroke in the older demographic does not indicate that the pulse pressure is unchanged in this group. Although the second case of a fixed pulse pressure is less likely, the similar results in both cases suggest that variations in MAP primarily account for differences in oxygen levels and perfusion following MCA occlusion.

Figure 6 explores the effects of different degrees of arterial stiffness (i.e., different distensibility) on the pulsatility of blood flow in the arteries and microcirculation since aging is associated with increases in arterial stiffness [45]. The higher distensibility value reduces the amplitude of oscillations in the time‐dependent pressure solutions by up to 83%, particularly in the arteries, arterioles, and capillaries, whereas the lower distensibility produces more variation in these pressures. This trend supports the idea that decreased elasticity and distensibility (increased stiffness) in arteries impedes their ability to stabilize flow delivered to the microcirculation [6], which results in higher variability in blood flow to these regions. These increases in flow variation and pulse pressure are considered detrimental as they are associated with ischemic stroke and even viewed as a predictive risk factor for cardiovascular events [5].

Figures 7 and 8 show how the time dynamics of pressure and blood flow change along the middle‐left region of the circulation, as well as how these dynamics are affected by both occlusion and an increase in MAP. In the distal microcirculation, there is less of a difference in the pressure waves corresponding to the varying MAP levels, indicating that the arteries upstream regularize variations in pressure. In the capillary compartment specifically, the difference between the peaks of the blood flow versus time waves corresponding to the control and elevated MAP values is 0.27 cm^3^/s in the non‐occluded case compared to 0.95 cm^3^/s with occlusion. The dynamics of the pressure waves near the capillary compartment at $P_{3,\mathrm{ml}}$ exhibit the same pattern in which the curve corresponding to the higher MAP lies further above the control MAP curve in the occluded case. This observation suggests that the effect of elevated arterial pressure is more pronounced when there is an occlusion and supports the idea that lowering MAP could have potential negative consequences on perfusion to regions downstream of the occlusion. The amplitudes of some pressure and flow waves appear to be reduced in the occluded case, but this change does not necessarily imply more dampening of oscillations. If there are overall lower values of pressure and flow, the amplitude relative to the average value may be maintained.

Cerebral autoregulation, which is examined in Figure 9, has important implications on the prognosis of stroke beginning from the early stages. Monitoring and analyzing such hemodynamic quantities can aid in clinical decision‐making and enhance the outcomes of reperfusion therapies [45]. The impairment of cerebral autoregulation is observed in the range of pressures for which autoregulation typically occurs, since the shape of the middle curve in Figure 9 is altered so that the plateau in blood flow occurs at higher pressure values beginning around 140 mmHg. Although the curves for the posterior and anterior regions do not change in shape or concavity, the flow is significantly lower for pressure values above 140 mmHg. The difference between occluded and non‐occluded flow in these regions increases with further increases in pressure. Improved blood flow compensation in the middle region with high MAP values may potentially lead to flow being directed away from the other regions to the middle. This change in flow distribution causes the anterior and posterior to exhibit more deficits in flow between the occluded and non‐occluded cases for higher arterial pressures. However, this theory is not conclusive and needs to be verified with further investigation.

The results shown in Figure 9 demonstrate that with the addition of the pulsatile pressure boundary condition and capacitances in the arteries and veins, the system maintains autoregulation patterns consistent with previous modeling studies [27] and experimental studies that have observed impairment in cerebral autoregulation immediately following ischemic stroke [46, 47]. This consistency verifies that approximating blood flow to the brain using a constant pressure boundary condition is a reasonable assumption if studying healthy individuals exhibiting relatively constant vessel distensibility. However, this model was designed to study individuals at advanced age, which is associated with both arterial stiffening and a greatly increased risk of stroke [2, 14], and thus distensibility is assumed to be highly variable. Therefore, distensibility emerges as an important characteristic of arteries and veins in this model. Since distensibility can significantly affect the dynamics of blood pressures and flows, previous mathematical modeling studies have demonstrated that incorporating capacitance elements provides a more thorough representation of cerebral blood flow regulation that is consistent with physiological observations [4, 23]. Overall, given the context of the present study, distensibility is a factor that warrants careful consideration in this model.

Although earlier results indicate that blood flow restoration following MCA occlusion is enhanced with higher MAP values, hypertension in stroke patients is often seen as a negative prognostic factor leading to increased stroke severity and a worse clinical outcome. A potential hypothesis explaining this trend is that physiological adaptations to a higher‐pressure environment lead to a rightward shift of the autoregulation curve [45]. If the relationship between blood flow and pressure is altered in this manner, then an increase in pressure may result in the same, instead of higher, flow. Therefore, hypertension may only be associated with disadvantageous factors such as inward remodeling of vessels [44]. An important consideration in the interpretation of model results is that only the effects of acute hypertension are examined, which can be viewed as moving in the positive direction along the autoregulation curve, whereas chronic hypertension corresponds to a rightward shift of the entire curve. When autoregulation is impaired, sudden reductions in blood pressure can put patients at a high risk of negative consequences [48], which is explained by the approximately linear decrease in blood flow with a decrease in pressure within the region of impaired autoregulation. Although lowering blood pressure after MCA occlusion has already occurred may be detrimental, adaptations with chronic hypertension suggest that in the absence of stroke, hypertension needs to be treated and managed to reduce the risk of stroke and to minimize damage should it occur.

Other common comorbidities such as diabetes, chronic kidney disease, and heart failure are thought to be risk factors for stroke and to lead to worsened clinical outcomes due to their effects on cerebral hemodynamics. A few studies have suggested that cerebral autoregulation is altered in patients with type 2 diabetes. However, this is not a consistent finding and requires further investigation. Both chronic kidney disease and heart failure lead to reductions in cerebral perfusion which alters the dynamics of blood flow and autoregulation [45]. Furthermore, the activation of the sympathetic nervous system in heart failure can affect vascular tone and smooth muscle reactivity [49, 50, 51]. An interesting future application of the present model could involve adjusting components, for example the parameters governing vascular tone, to correspond to the differences in blood flow, autoregulation, and vessel smooth muscle tone associated with frequently comorbid conditions. Doing so may provide a clearer understanding of how these conditions result in worsened stroke severity and how this prognosis can be improved.

### 4.1 Model Limitations

To construct a model of the primary components of the cerebral circulation, simplifications were made to render the problem more tractable. For instance, the pressures used in the differential equations for diameter and activation are averages taken over the length of a period of arterial pressure. Given the significantly different time scales between pressure pulsatility (on the order of 1 s) and typical vessel dilation (on the order of 1 min), it is reasonable to assume that vessel dynamics depend on past average behavior. This assumption has been used effectively in many previous models. However, the precise method by which a vessel may average past behavior and use it to enact vessel dynamics is not fully known and is worth further consideration. In the current model, the effects of time‐dependent pressure variations on vessel mechanics may not be as prominent since the averaging used here stabilizes the pressure values used for the autoregulation calculations. The stimulus for these autoregulation calculations contains a metabolic signal that is determined by the partial pressure of oxygen, whereas other models have used a CO_2_‐dependent regulatory mechanism [26, 52, 53]. Although the present model did not explicitly incorporate or track CO_2_ levels, the use of an oxygen based conducted response signal is not unreasonable since levels of CO_2_ and oxygen are closely related. That said, given the importance of CO_2_ in cerebral blood flow regulation, future models could include both an oxygen and carbon dioxide dependent mechanism for flow regulation. Furthermore, capacitances were only considered for the arteries and veins because large vessels have the greatest elasticity and capacity to store flow, but smaller vessels such as the arterioles and venules could potentially have some ability to behave as capacitors and could be explored in future models.

Due to the compartmental nature of the model, simplifying assumptions were made regarding blood vessel geometry. Since vessels are represented using resistors arranged in series or parallel, the specific location and orientation of the individual vessels in three‐dimensional space is not modeled. The measure of position in Figures 3 and 5 reflects the sum of the lengths of the vessels along the pathways shown in Figure 1, not the precise physical location in the vasculature. The model geometry posed some uncertainties about the calculation of the conducted response signal. The behavior of metabolic signaling at three‐way bifurcations, such as point $P_{2,\mathrm{ml}}$ (see Figure 1), has not been previously studied. In addition, blood flow in collateral vessels could potentially be bidirectional. For example, flow in the COL_aml_ could be directed from the anterior towards the middle branch (from $P_{2,\mathrm{al}}$ to $P_{2,\mathrm{ml}}$) or in the opposite direction depending on the pressure gradient between the endpoints $P_{2,\mathrm{al}}$ and $P_{2,\mathrm{ml}}$. To account for these types of possibilities, the conducted response signal had to be adapted to allow for propagation in two different directions.

## 5 Conclusions

This study reveals how changes in incoming arterial pressure and vessel distensibility affect compensatory responses to ischemic stroke caused by MCA occlusion. Due to disturbances in autoregulation with occlusion, pressure boundary conditions corresponding to elevated MAP can have protective effects by significantly reducing the degree of ischemia in dependent tissue regions. The present study also explores how variations in arterial stiffness can impact the patterns of pressure dynamics at locations of the cerebral circulation both proximal and distal to the occlusion, as well as assesses the ability of the cerebral circulation to accommodate pulsatile flow under both occluded and non‐occluded circumstances. The model results offer valuable insight into not only the impact of stroke on the microcirculation, but also how changes in relevant factors such as arterial distensibility and the incoming pressure boundary condition combine with the consequences of the occlusion. This information applies to a wide demographic of stroke patients since conditions that increase arterial stiffness such as hypertension and increasing age are also considered risk factors for stroke. The model also has the potential to be adapted to other physiological conditions by accounting for more specific adaptations such as changes in the thickness of the vessel wall that occur with chronic hypertension [54]. Thereby, this study lays the foundation for future work exploring the relationships between vessel compliance, arterial blood pressure, and cerebrovascular conditions.

## 6 Perspectives

The mathematical model of the cerebral circulation integrates vascular compliance, pulsatile flow, and flow regulation mechanisms to simulate cerebral hemodynamics under ischemic stroke conditions. By expanding this model to account for the distensibility of larger vessels and their ability to accommodate pulsatile flow using capacitance elements, the model simulations yield more accurate predictions of physiological behaviors that align with experimental data. This vascular model provides improved predictions of cerebral tissue oxygenation following middle cerebral arterial occlusion and may be useful in assessing early stage interventions in patients at high risk for stroke.

## Author Contributions

E.Z. contributed to the writing, reviewing and editing of the manuscript, development of computer code and all model simulations, model analysis and interpretation. J.B. contributed to the reviewing and editing of the manuscript, supervision, visualization, and model analysis/interpretation. S.S.M.‐S. contributed to the conceptualization and reviewing and editing of the manuscript. S.K. contributed to the conceptualization and interpretation of the study. C.K.S. contributed to the conceptualization, investigation, and reviewing and editing of the manuscript. J.A. contributed to the writing, reviewing and editing of the manuscript, investigation, conceptualization, methodology, project administration, supervision, visualization, and model analysis and interpretation.

## Funding

J.A. gratefully acknowledges NSF DMS‐1654019 and NIH R01EY030851. J.A. and J.B. gratefully acknowledge NSF DMS‐1852146 and NSF DMS‐2150108. J.B. gratefully acknowledges NSF DMS‐1951531. C.K.S. and S.S.M.‐S. gratefully acknowledge R01NS042617.

## Ethics Statement

All ethical standards of research were upheld.

## Conflicts of Interest

The authors declare no conflicts of interest.

## Data Availability

Data and computer code will be made available upon request to the corresponding author.
